# Advanced high resolution three-dimensional imaging to visualize the cerebral neurovascular network in stroke

**DOI:** 10.7150/ijbs.64373

**Published:** 2022-01-01

**Authors:** Chudai Zeng, Zhuohui Chen, Haojun Yang, Yishu Fan, Lujing Fei, Xinghang Chen, Mengqi Zhang

**Affiliations:** 1 Department of Neurology, Xiangya Hospital of Central South University, Changsha, Hunan, China, 410008; 2 National Clinical Research Center for Geriatric Disorders, Xiangya Hospital, Central South University, Changsha, China, 410008

**Keywords:** High resolution 3D imaging, Stroke, Neurovascular network, Synchrotron radiation, Two-photon microscopy, Photoacoustic imaging, Magnetic resonance imaging, Light-sheet microscopy

## Abstract

As an important method to accurately and timely diagnose stroke and study physiological characteristics and pathological mechanism in it, imaging technology has gone through more than a century of iteration. The interaction of cells densely packed in the brain is three-dimensional (3D), but the flat images brought by traditional visualization methods show only a few cells and ignore connections outside the slices. The increased resolution allows for a more microscopic and underlying view. Today's intuitive 3D imagings of micron or even nanometer scale are showing its essentiality in stroke. In recent years, 3D imaging technology has gained rapid development. With the overhaul of imaging mediums and the innovation of imaging mode, the resolution has been significantly improved, endowing researchers with the capability of holistic observation of a large volume, real-time monitoring of tiny voxels, and quantitative measurement of spatial parameters. In this review, we will summarize the current methods of high-resolution 3D imaging applied in stroke.

## 1. Introduction

Stroke is an ischemic or hemorrhagic disease mainly occurring in the cerebral microvascular system, leading to injury of cerebral vasculature and irreversible damage to the neurological function and even to death 1. The importance of better understanding of its pathogenesis to help find more effective treatments cannot be overlooked. However, neurons that stretch in multiple directions and blood vessels make up a complex spatial network. The research of stroke is no longer satisfied with the information only on two-dimensional planes. Their nature can only be demonstrated in three-dimensional (3D) imaging. Thus, an advanced 3D brain atlas is released as an assist for the study of neural 3D structures 2. Meanwhile, keeping the tight and orchestrated neurovascular relationship between nerves and blood vessels is the basis for maintaining normal physiological activities of the brain. In stroke, a specific association will emerge. For example, angiogenesis after vascular injury depends on repair-associated VEGF-expressing microglia 3, and endothelial cells of cerebral vessels secrete trophic factors that can be directly neuroprotective 4.

Therefore, the neural networks, cerebrovascular networks, and interaction between them have become an intriguing subject of study for stroke. As noted above, the temporal and spatial studies using microscopic observation methods with multiple resolutions (from the nanoscale to the microscale) provides new insights beyond perspectives from biochemical analysis and genetic testing. Biochemical analyses such as gel electrophoresis and chromatography, and genetic tests such as polymerase chain reaction can quantitatively or qualitatively understand regional effector molecules to explain pathological changes, but lack visualization of spatial distribution and relationships. Thus, the intricate spatial interaction of neurons and vessels is unobtained. For decades, microscopes designed for visualization naturally shift their focus from flat to spatial relationships, and even to spatial dynamics, leading to the development of the imaging from 2D to 3D and even 4D (3D + time). Wang K et al. simultaneously visualized the flow of blood cells through thousands of blood vessels in the cortex of awake mice 5.

Take the study of blood vessels as an example. Dysfunction of small blood vessels is an important process of stroke, which often includes changes in cerebral blood flow and vascular structure 6, 7. Histological studies are commonly employed for standard analysis of biological specimens. However, histological analysis as a two-dimensional technique has some inherent limitations: the 3D structural integrity of the brain is destructed by the sectioning, bias or error may also occur in slice selection which is based on the assumption that the sample is relatively homogeneous, and sectional observation is discontinuous and lacks description of spatial relations 8, 9. And the view of the electron microscope is too small. These disadvantages have led researchers to call for the utilization of high-resolution 3D imaging and stimulated the development of the higher resolution of magnetic resonance imaging (MRI), the two-photon microscope, the computer imaging model, and suchlike 10.

The development of the microscope closely followed the improvement of resolution. Microscopy and ultrahigh-resolution microscopy techniques do help explain the brain at the nanoscale micro level, which is used to discover new intracellular structures, analyze biomolecular interactions, and quantify molecular counts 11. Representative work was that Zhuang et al. analyzed the membrane-associated periodic skeleton in nerve cells and observed that the ring structure of actin formed a periodic structure with an interval of 180-190 nm 12. However, at the micron level (1-10 μm), a 3D imaging platform is still needed to solve the spatial relationship between hard and soft tissue and provide 3D quantitative characterization 13. The use of new imaging mediums, like synchrotron radiation, or new modes, like light-sheet microscopy has greatly improved the resolution of 3D imaging. Based on different methods, including light, acoustics, and nuclear magnetic resonance, high-resolution 3D imaging technology is gradually being applied to stroke to investigate cerebral refined structure, function, and metabolic status, etc.

## 2. Light

### Synchrotron Radiation

SR's wide spectrum ranges from infrared to hard x-rays. X-rays, which are the most commonly used SR source in biomedicine, make better use of imaging with smaller wavelengths and greater penetration. This advantage is enhanced by synchrotron radiation (SR) X-ray which is the electromagnetic wave emitted along the tangent direction of the orbit when the magnetic field of the storage ring changes the motion direction of electrons at sub-light speeds. It has the characteristics of high intensity, monochromaticity, collimating coherence, and high spatial resolution around 1,000 times higher than that of conventional X-Ray absorption imaging 14. There are currently more than 20 synchrotrons worldwide 15. The third generation of high-performance light sources consists of two types: one with high electron energy (6 GeV), such as American Light Source (APS) 16, and the European Synchrotron Radiation Facility (ESRF) 17, another with medium electronic energy (2-3 GeV) such as Swiss Light Source (SLS) 18, and Shanghai Synchrotron Radiation Facility (SSRF) 19. Based on those, synchrotron radiation light source was developed to the fourth generation of free-electron laser light source, like Swedish Max-Ⅳ 20. But third-generation sources are more commonly used, such as SSRF which produces X-rays from the electronic storage ring with an acceleration energy of 3.5 GeV and an average beam of 200 mA. With a tunable energy range of about 8-70kev, the resolution eventually reaches micron-scale or even sub-micron scale 21, 22.

As the importance of brain microvasculature in stroke, SR has provided a mighty impetus in the study of stroke 14. Conventional SR uses absorption angiography, including K‐edge subtraction angiography (KESA) for large structures and single‐energy temporal subtraction (SETS) for higher resolution 21, 23, 24. Phase contrast imaging (PCI) can detect micron or submicron microvessels without angiography, which can be divided into inline X-ray phase contrast imaging (ILPCI), grating interferometry (GI), and crystal interferometry (CI), etc 25, 26.

3D imaging using absorption angiography is mainly used in vivo. Immunohistochemical staining can only show the number of cerebral vessels rather than the number of perfusion vessels, but SR can realize dynamic observation of brain microvessels 27. Yang G et al. continuously achieved the dynamic detection and assessment of thrombolysis and recanalization at a 9μm resolution in rats 28.

**Figure 1:**
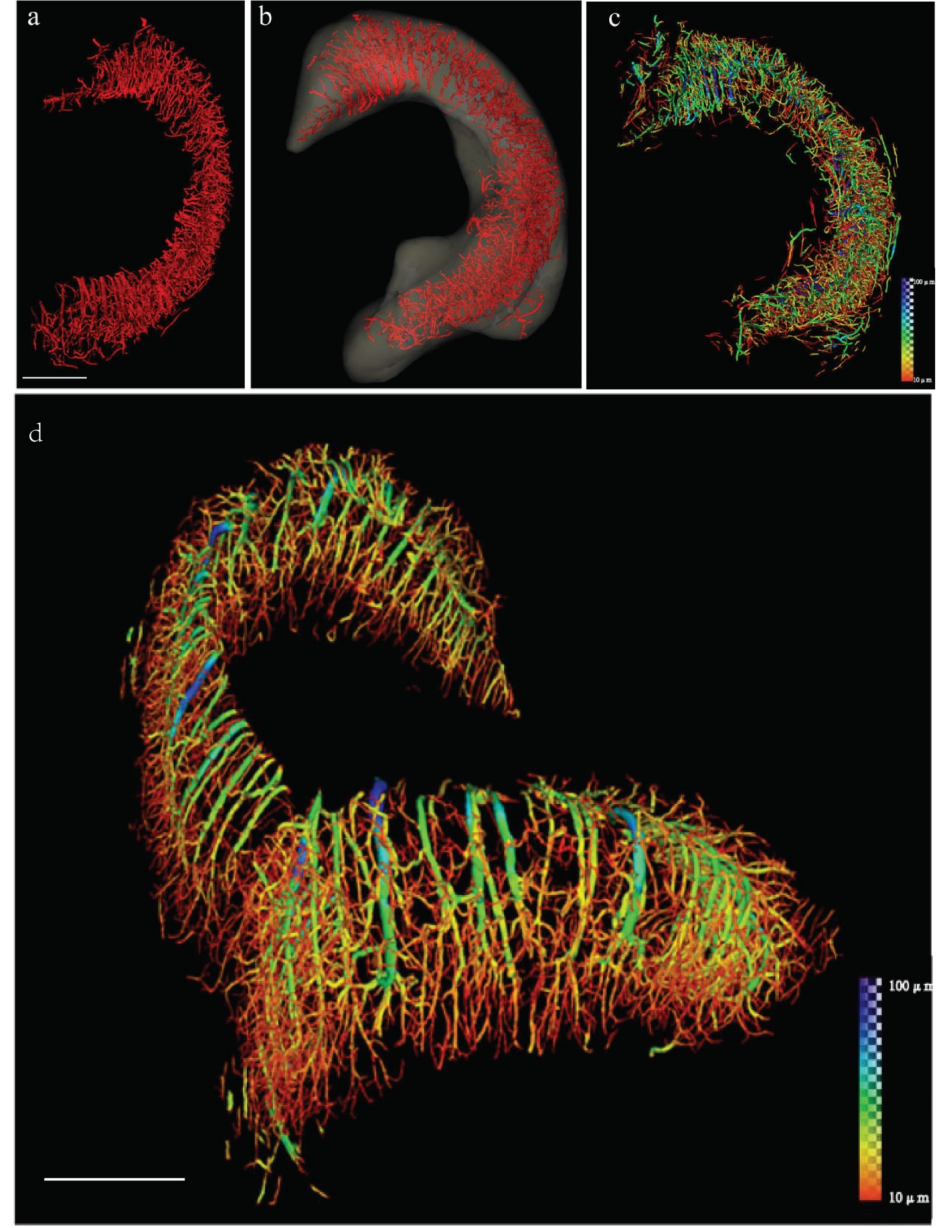
Reconstruction of the 3D angioarchitecture of the hippocampus in normal rat brain by SR. (a, b) By SR-ILPCI reconstruction, the spatial structure distribution of the right hippocampal three-dimensional network was extracted from the whole brain (a) and accurately outlined (b) (scale bar, 600 μm). (c) Hippocampal vessel diameter rendering identifies the distribution of vessels with different diameters. (d) The original distribution of intracerebral vessels within the hippocampus with continuous pseudocolor changes depicting the distribution spectrum of vessel diameters, ranging from 10 μm to 100 μm (dark blue) (scale bar, 700 μm). Reproduced with permission from Reference 34.

Phase contrast 3D imaging is mostly used in vitro, which is more suitable for neurovascular networks in stroke, and can provide more multi-dimensional and multi-scale information without contrast agents compared with absorption imaging 29. Unlike absorption contrast imaging (ACI) where incomplete filling or leakage of the contrast agent will inevitably affect the quality of the image obtained 30, PCI shows good applicability for vascular structure 31, 32. In addition to vascular structure and hemodynamics, PCI can also be used for semiquantitative studies on angiogenesis. It was found that the perfusion vascular density in the area around the ischemic infarction of middle cerebral artery occlusion (MCAO) rats treated with Dl-3-N-butylphthalide was higher than that of the silicone oil group, indicating that butylphthalide treatment promoted angiogenesis in the area around MCAO infarction 33. In MCAO mice, ILPCI was used to reconstruct the 3D vascular structure, showing that the vascular trees are arranged in a tangled pattern to form a network, identifying vessels with diameters of about 11.8 microns 14. Epilepsy also presents with microvascular changes like stroke, SR 3D imaging was used to measure microvessel parameters, including vessel surface area, number of vascular segments, and number of vascular bifurcations, with a pixel size of 5.2 μm **(Figure 1)** 34. Combined with microcomputed tomography (μCT), SR was used to detect the three-dimensional structural changes of brain microvessels in rats 4 hours, 6 hours, 3 days, and 18 days after ischemia 22.

For nerves, Golgi-cox staining is a basic method 35. However, based on changes in electron density, Mareike Topperwien et al. successfully visualized and automatically located about 10^6^ nerves in different layers of the cerebellum without any additional staining 36. Fonseca MC et al. combined μCT SR with Golgi-Cox mercury-based impregnation protocol in the brain of mice, achieving an adjustable pixel size of 0.82 μm for high resolution/small field of view (FOV) imaging without tissue slicing or clearing 37.

At present, SR 3D imaging is still mostly used in microvascular studies in vitro, but the interaction between microvascular network and nervous system in stroke is inevitable in imaging. The imaging of soft tissues by using no X-ray contrast agent can be easily redirected and resegmented virtually in a computer with a resolution of 5-10 μm 38. The update of μCT allows scientists to identify neurons, glial cells, and vascular systems in the 3D imaging of SR, and effectively measures the cell spacing and distance between cells and blood vessels 39. Anna Khimchenko et al. used hard X-ray in ESRF to make the effective pixel size up to 130nm, which can distinguish subcellular structures such as nuclear membrane and nuclear pore of Purkinje cells 40. However, improving the resolution to observe subcellular structures necessarily confines the sample to the micron scale and takes time to match the sample's anatomical markers 9.

In addition to the 3D reconstruction of microvascular network and nervous system, multiple other techniques based on SR can be combined to capture various types of data in a high-resolution manner. For instance, SR-infrared spectrum (SR-IR) or SR micro-X-ray fluorescence (SR-μXRF) can observe the biochemical properties of neurons and vessels. Metabolic changes occurring in neurovascular network are markers of stroke 41. SR-IR is 100-1000 times brighter than the traditional light source, enabling fourier transform infrared spectro-microtomography (FTIR-μCT) to reconstruct the image in the micron scale. By SR X-ray correction 42, 43, IR was used to construct a 3D mapping of *in-situ* content of glucose, glycogen, and lactate in the brain of mice 44. Transition metal homeostasis, such as copper, iron, and zinc, is an important property of brain and plays an important role in stroke and other brain diseases 45-47. As studies on ion distribution in stroke increase gradually like post-stroke neuroplasticity or ischemic stroke, SR-μXRF has become a powerful technique for ion distribution in biological samples by utilizing the secondary characteristic radiation induced by X-ray 48, 49. Although not yet thoroughly used in the brain 3D imaging, SR-μXRF will show great potential in the field of stroke.

### Two-photon/Multi-photon microscopy

Based on the nonlinear optical microscope driven by the high peak power that is possible with ultrashort pulses, the two-photon microscopy (TPM) first appeared in 1990, invented by Webb, etc 50. TPM uses near-infrared (NIR) light instead of the visible or ultraviolet light used by conventional laser confocal microscopy. Under the high photonic flux required by the nonlinear optical process, the fluorescent molecule absorbs two long-wavelength photons at the same time, and then emits a photon with a shorter wavelength after reaching the excited state, achieving imaging. Because two-photon or multi-photon imaging requires high photon density of exciting light, only fluorescent molecules in very small volumes in and around the focal point can be excited, which enables TPM and multi-photon microscopy (MPM) to have very high three-dimensional resolution 51. Currently, a fast high-resolution two-photon microscope invented by Cheng H's team can achieve a horizontal resolution of 0.64 μm, an axial resolution of 3.35 μm, and an imaging frame frequency of 40 Hz 52. At the same time, short pulses with longer wavelengths are used to allow two or more atoms to be absorbed to reduce scattering and achieve deeper imaging 53. Compared with conventional laser confocal microscopy, the n-photon absorption probability decreases rapidly, thus photobleaching and phototoxicity occur only at the focus 54. Other variations were also developed like third-harmonic generation 55.

**Figure 2:**
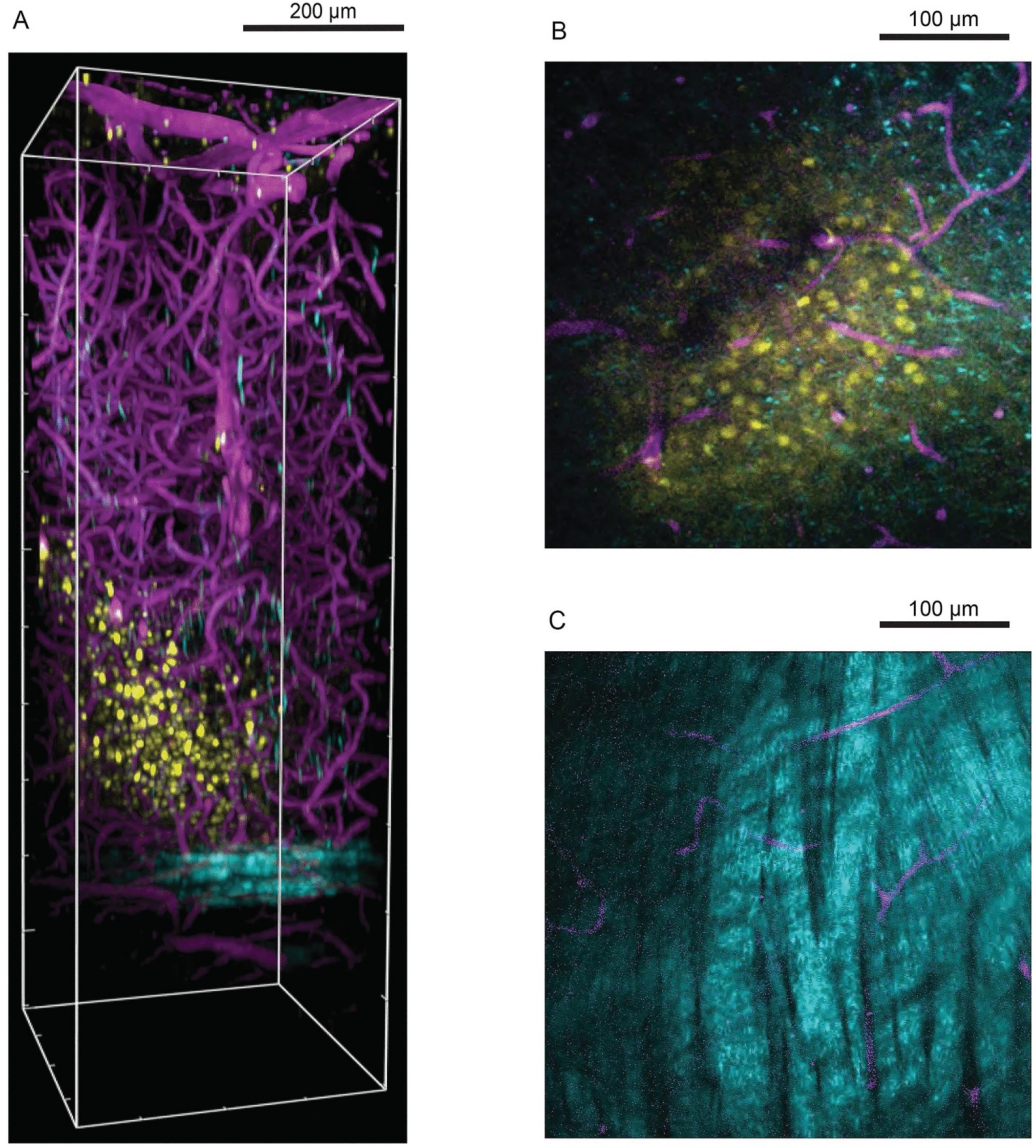
Three-photon microscopy in visualizing cells deep in mouse visual cortex relative to blood vessels and white matter. (A) Side view perspective of a three-dimensional reconstruction of the three-photon image volume depicting the field of cells with respect to blood vessels and the white matter. Oregon Green labelled cells in layers 5-6 are shown in yellow, blood vessels labelled with Texas Red Dextran are shown in magenta and cortical white matter imaged with THG is shown in cyan. x-y-z volume dimensions: 375 µm × 375 µm × 1100 µm. (B) Single z plane three-photon image at 750 µm below brain surface showing labelled cells and blood vessels. (C) Single z plane three-photon image in the white matter at 900 µm below brain surface. Reproduced with permission from Reference 67.

The application of TPM in stroke is primarily to explore cerebral information in live behaving mammals, The confocal laser scanning microscope allows to scan structures about 100 mm below the surface, but the imaging of structures such as the neuron group requires deeper penetration, about 300-500 micrometers below the surface. TPM/MPM can alleviate this problem by providing high-resolution, non-invasive, deep tissue observations 56. But to scan the brain, the covered opaque skull must be thinned down to a thickness of 15 µm or partially removed to form a cranial window 57. Subcortical imaging of the neocortex can now be achieved by improving coverslip to minimize the effect of spherical aberration 58. The methods of fluorochromes administration include intravenous delivery, electroporation, and virus transduction; and for cerebrovascular labeling, the intravenous delivery of a fluorescent dye can efficiently stain an entire vascular tree from the organ 59. The detection of neuronal activity is necessary for the studies of injured brain, so the TPM/MPM applied to neurons in stroke is mostly functional. Calcium imaging is an essential tool for neural imaging 60. TPM calcium imaging was used to study how microglia influence neuronal activity after stroke 61. Lamiae Abdeladim et al. designed a chromatic multiphoton serial (ChroMS) microscope to provide high-contrast multicolor imaging and tracked 261 chromophores formed by 1055 astrocytes in the 1.2×2×1 mm^3^ area, with the voxel size of only 0.54×0.54×1.5 µm^3^ 62. In addition to calcium ions, TPM can also visualize the distribution of magnesium, zinc, and nickel ions 63, 64. For deeper parts, with a two-photon absorption fluorescent probe, microvessels more than 1mm deep beneath the skin can be imaged in three dimensions 65. Gradient-index (GRIN) fiber probe implants can be used to go much deeper, distinguishing synapses in the hypothalamus that are 5mm deep^66^. MPM enabled high-resolution imaging in deeper brain areas **(Figure 2)** 67. Chris Xu et al. successfully used a three-photon microscope to image the vascular structure in the hippocampus of mice and neurons labeled with red fluorescent protein, with a resolution of about 4.4μm at a depth of about 900 μm 68.

Another characteristic application of TPM is the miniaturization of free-moving mice. It makes up for the absence of a benchtop microscope in the study of long duration behavior or social behavior. Baris N. Ozbay et al. used head-mounted miniature TPM to observe about 200 oligodendrocytes labeled with a green fluorescent protein in the FOV with a diameter of 240μm, with a transverse resolution of 1.8 μm and an axial resolution of 10 μm 69. However, the miniaturization of TPM still requires effective fluorescence collection, compact and fast scanning mechanisms, and reduction of motion artifacts 70. Recently, Angus Silver's team achieved real-time movement-corrected 3D two-photon imaging with submicrometer precision 71.

It is worth mentioning that wavefront distortions constrict the achievable resolution with penetration depth, but adaptive optics systems (AOS) enable wavefront correction. Meanwhile, it can reduce the power requirement for two-photon imaging of brain activity in awake mice by more than 30 times 72. AOS was applied to high-speed 3D dynamic imaging of calcium, blood vasculature, microglia, and structure of the neuronal network in vivo mammalian brain 73. TPM/MPM is still limited by the speed at which scanning can be realized. Multi-focal excitation generated by adaptive optics is an improved method 74.

### Light-sheet microscopy

Due to the photobleaching and phototoxic effects of excitation light, most optical imaging modes cannot achieve the long-term observation of living cells and the recording of intracellular life activities 75. The light-sheet microscopy (LSM) mode explained by Ernst Stelzer has become an important turning point in the development of imaging technology. It uses excitation and detection objectives arranged at right angles to form layered light and separate the excitation and emission light paths to ensure selective excitation of specific planes; thus it reduces ineffective exposure, photobleaching, and phototoxicity 76. In 2004, a light sheet microscope named Selective Plane Illumination Microscope (SPIM) was formally born. It is widely used for 3D imaging of large samples due to its huge advantage of shortening imaging time and reducing phototoxicity 77. Later, scientists are seeking a resolution that meets both the large FOV and the diffraction limit close to the Rayleigh Abbe rate 78, 79. The thinner a Gaussian beam is, the higher the axial resolution is. The Gaussian beam whose cross-section radius track is hyperbolic makes the area with a relatively thin thickness limited, which means that if the light sheet is thin, the light sheet is short^80^. It means that it is difficult to have both the FOV and the resolution. In contrast to the Gaussian beam, Bessel beam has no diffraction and low phototoxicity, enabling faster acquisition speeds and higher signal-to-noise ratios. Betzig Lab first applied them to light-sheet microscopes, resulting in an isotropic resolution of 300 nm 81. Later, the lattice light-sheet microscope (LLSM) used a spatial light modulator to adjust the shape of the beam entering the illumination objective to multi-beam Bessel interference, creating an optical lattice, and finally forming an ultra-thin light sheet. It makes it possible to achieve high spatiotemporal resolution, large FOV, and low phototoxicity 82. Multi-view imaging is implemented in diSPIM and isoView, and becomes a potential candidate for high-volume high-resolution imaging 83, 84. For example, isoView microscopy can achieve an isotropic spatial resolution of 400 nm, and the volume throughput per second is greater than 10^8^ µm^3^ 84. Two-photon excitation allows them to reach greater depths 85.

LSM is limited by artifacts when used in large tissue of stroke brains. For example, if the sample contains high-density structures such as pigments that block excitation light, or lipids that cause light scattering and uneven illumination, it will result in some dark lines. Tissue removal is currently a common solution. Its effect of improving penetrating power and reducing light scattering is well adapted to the three-dimensional imaging of brain tissues, especially the whole brain so that people can obtain volume images of the entire brain without physical slicing. It should be noted that the tissue removal technique is not limited to LSM, but is used in a variety of microscopy techniques such as MRI 86. The earliest attempt was to use a mixture of benzyl alcohol and benzyl benzoate (BABB/Murray's clear) to process the specimen to observe the brain neural network, and at present, the commonly used tissue removal techniques can be divided into three methods: hydrophobic, hydrophilic and hydrogel-based 87. For example, CLARITY 88 and DISCO 89 can obtain sub-cell-level resolution. Samples with or without tissue removal are most commonly embedded in agarose or placed in a liquid-filled chamber, but not limited to this 90.

The LSM played a great role in imaging the structure and function of nerves in stroke. It enables researchers to perform whole-brain functional imaging and optogenetics research at a single-cell resolution 91, 92. Observation objects changed from rodents to humans 93. The use of ultra-microscopy beyond the diffraction limit will increase the resolution by nanometers. The joint use of expansion microscopy (ExM) and LLSM can visualize and quantify nerve groups, subcellular structures, and synaptic connections with a resolution of 60 × 60 × 90 nm^3^ 94. And the lateral average accuracy obtained by combining single-molecule positioning technology can reach 7.2 nm 95. Non-invasive research has always been the goal. Swept confocally-aligned planar excitation (SCAPE) microscopy through the cranial window can monitor neuronal network activity in the brain of awake and freely moving mice, and distinguish single capillaries of 5-10 μm in depth at 140 μm 96. And SCAPE2.0 penetration depth can reach 450 μm 97. Near-Infrared II Window LSM has further realized the live imaging of the head in depth at 750 μm 98.

**Figure 3:**
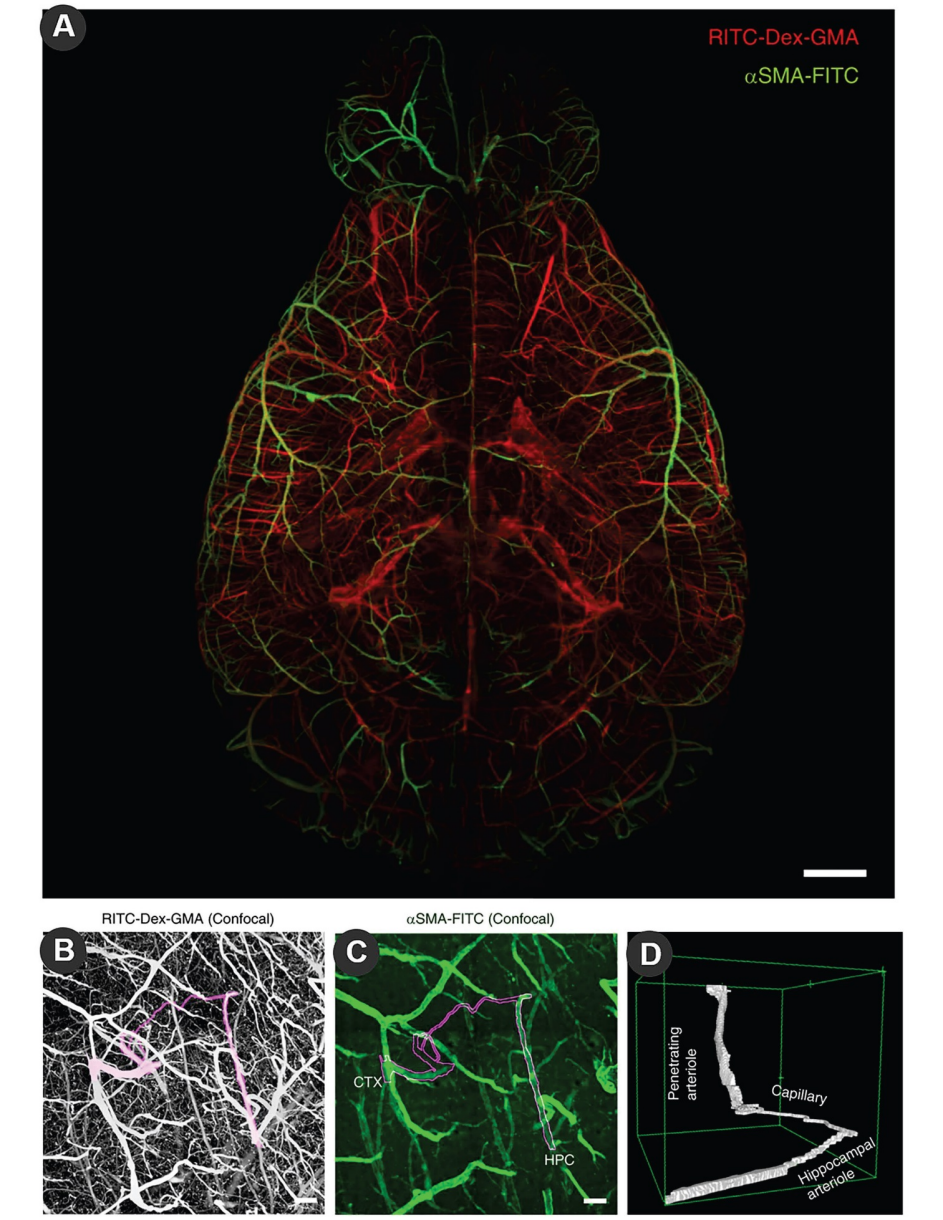
Large-scale molecular phenotyping-compatible 3D imaging of cerebral vasculature. (A) Maximum projection of light-sheet fluorescence microscopic images of the entire vasculature in a SeeNet-treated brain (1.6× zoom, ×0.63 objective lens, numerical aperture: 0.15, working distance: 87 mm) into a single stacked photo. Casted vessels are shown in red. Arterioles immunolabeled with anti-α smooth muscle actin (anti-αSMA) are shown in green. Scale bar = 1 mm. (B) Maximum projection of confocal images of casted vasculatures from the pia to the hippocampus. A cortico-hippocampal vascular path is shown in magenta. Scale bar = 100 μm. (C) The anti-αSMA immunosignal in the same field is shown on a green scale. Scale bar = 100 μm. (D) A 3D-rendered view of the cortico-hippocampal vascular path. Reproduced with permission from Reference 104.

The vasculature of the brain can also take full advantage of LSM through dye filling strategies, such as the special shape of cortical microvessels 99, and changes in blood vessels after cerebral ischemia 100. The introduction of tissue removal provides three-dimensional imaging of the vasculature. TubeMap designed by Nicolas Renier uses iDISCO to construct, analyze and visualize the blood vessel map with micron resolution containing 100 million blood vessel segments 101. Fluorescein isothiocyanate (FITC)-conjugated albumin was injected with light film microscopy to assess large-scale microvessels in the ischemic brain 102. Jonas Gregorius et al. determined microvascular network characteristics after focal cerebral ischemia with a resolution of 2 µm, including microvascular length density, branching point density and microvascular tortuosity 103. In order to reduce the false negatives of vascular imaging of conventional molecular characterization, the newly developed SeeNet method improves the dye to enable three-dimensional imaging of almost whole brain microvessels** (Figure 3)** 104.

Simultaneous three-dimensional imaging of neurons and vessels is an important development direction for stroke. LSM can simultaneously study qualitatively and quantitatively neurovascular networks 105. So far it has been used in neurodegeneration and neurodevelopment 106, 107.

In the existing high-resolution large-sample imaging methods, it is a common solution, like IsoView LSM, to use a tiling strategy to reconstruct 3D samples of the entire nervous system, but it also means complex graphics processing algorithm 108. Better strategies are being studied, such as the axially swept light-sheet microscopy, which maintains an isotropic resolution of 390 nm over a larger FOV 109. In addition, a multiangle-resolved subvoxel selective plane illumination microscope (Mars-SPIM) can collect an atlas of the entire mouse brain in 30 minutes with an isotropic resolution of 2μm 110. A new algorithm for LSM devised by Shroff H's team realized imaging processing time ten-fold to several thousand-fold faster compared to previous methods 111. Of course, LSM still needs technology that can image large samples with a co-directional resolution of less than 1μm and a faster and more accurate algorithm for processing data sets over tens of megabytes 87.

### Imaging System Combined Serial sectioning

Accurate observations of deep nerves and vessels take an increasingly integral role in stroke, and then a new idea boldly alternates imaging with slicing, overcoming depth to produce high-resolution 3D images of the whole brain. Micro-optical sectioning tomography (MOST) devised by Luo Q et al. is a new type of neural optical imaging system, which can simultaneously scan ultra-thin slices processed by diamond cutter for optical imaging and obtain sub-micron brain data with high throughput 112, 113. When the thickness of the tissue slice is less than the optical diffraction limit, the axial resolution is the slice thickness, and the transverse resolution is determined by the optical imaging configuration. MOST allows 3D imaging of neurons and neuronal connections to reconstruct a digital mouse brain with a voxel size of 0.33×0.33×1.0 μm^3^ 112. 3D representation of cerebrovascular network also shows great advantages in quantitative analysis of microvascular distribution at the resolution of micron 114. The improved Nissl staining method can simultaneously image brain cells and angiography at a resolution of 1 micron voxel 115. MOST can obtain a voxel resolution of 0.35 × 0.4 × 1 µm in MCAO mice to reconstruct microvasculature 116. The combination of rabies virus markers with fluorescent MOST (fMOST) can show the input of single neurons in the whole brain, providing a reliable option for optogenetics. The imaging speed was also improved, and the entire mouse brain could be imaged in an hour at a voxel size of 1.30×1.30×0.92 μm^3^ with a section thickness of 40 μm, without optical clearance 117. The drawback is that the data set is too large and thus makes data processing more difficult, but this is the inevitable result of this kind of idea.

**Figure 4:**
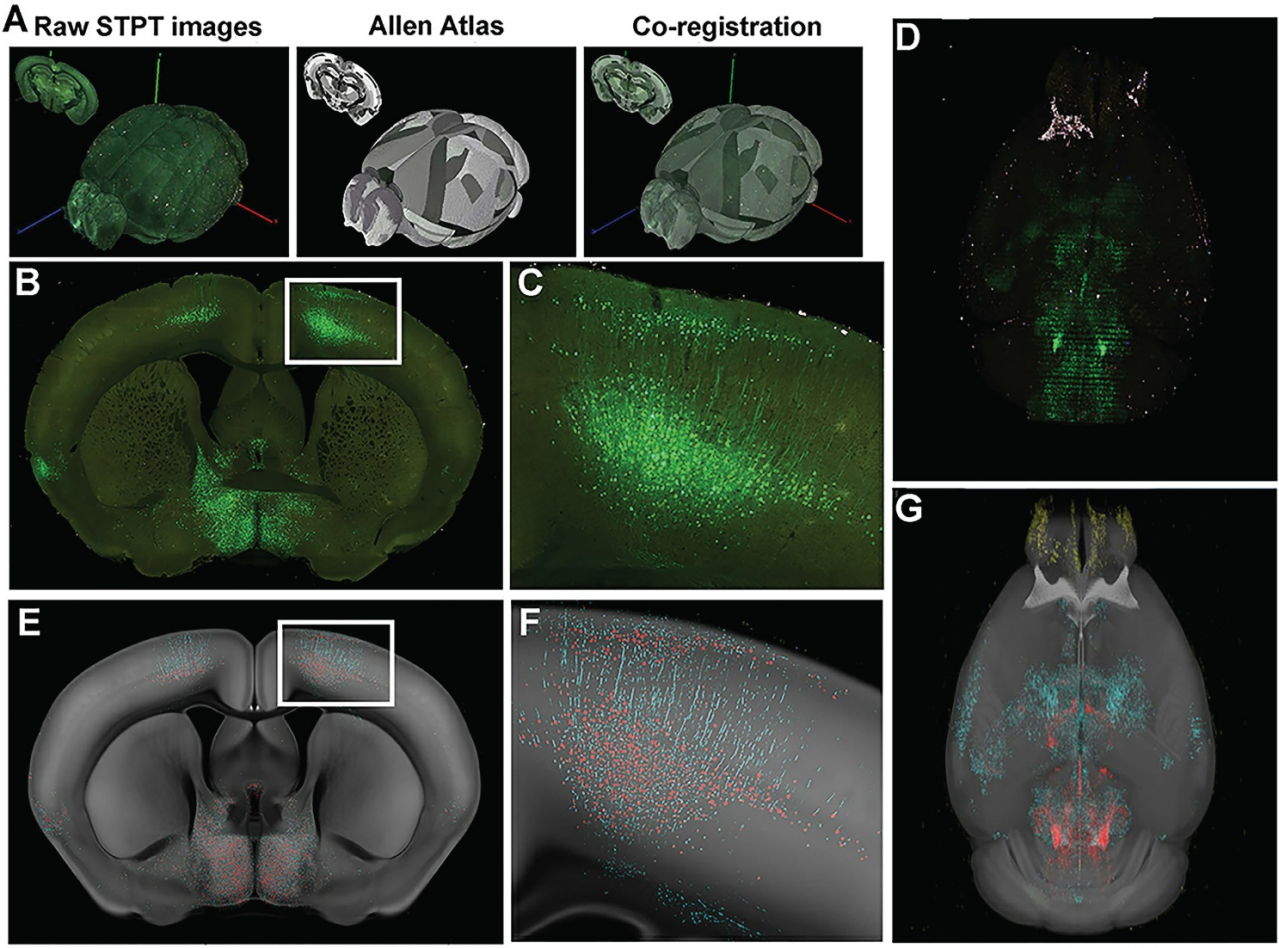
Serial two-photon tomography of the whole mouse brain. (A) Overview of STP data acquisition and registration. (B) Representative coronal section raw fluorescence image, with (C) expansion of areas identified in white box in B showing green fluorescent protein (GFP)+ neuronal cell bodies and processes. (D) Coronal images are stacked into a 3D representation and co-registered to the Common Coordinate Framework (CCF 3.0; Allen Institute for Brain Science) Atlas. (E) Probability map output of the same section shown in (B) depicting pixels classified via machine learning (ML) as either GFP+ cell bodies (red) or GFP+ neuronal processes (cyan) overlaid onto the corresponding section from the atlas (CCF 3.0 average template) shown in gray. (F) Enlarged image of boxed area in (E) showing fine detail and accurate classification of neuronal cell bodies and processes corresponding to the raw fluorescence image shown in (C). (G) 3-D rendering of the probability maps corresponding to the raw fluorescence images of the brain shown in D. GFP^+^ cell bodies, red, GFP^+^ neuronal processes, cyan, atlas images, gray. Reproduced with permission from Reference 122.

In fact, slicing and imaging combined with new strategy still show vast potential, and multiple techniques appeared based on this concept, such as serial two-photon tomography (STP), block-face serial microscopy (FAST). The resolution of FAST can reach 0.7×0.7×5 μm^3^, and the images are collected at z-sampling intervals of 50-100μm; its rapid imaging ability reduces the time of imaging the mouse brain to 2.4 hours^118^, ^119^. Mereuta OM et al. analyzed ultrastructure of clots in acute ischemic stroke at a resolution of 50 nm with FAST 120. STP can also conduct 3D imaging of the brain at the resolution of micron and submicron, and avoid embedding the tissue to weaken the intensity of fluorescence signal 121. It facilitates the construction of 3D neural connections 13. Later studies were more in-depth. For example, Stowe AM et al. used STP to track CD8^+^T cells to visualize the whole brain of post-stroke neuroinflammation **(Figure 4)** 122, and monitor the migration of B cells to visualize neurogenesis and functional recovery after stroke 123. Deep learning can help to reconstruct the 3D blood vessel and white matter network at the same time 124. However, STP also has manifest disadvantages. It needs to express the components of fluorescence and imaging is too time-consuming 125.

### Optical coherence tomography

Optical coherence tomograph (OCT) is an intravascular imaging modality that utilizes near-infrared light to generate images by interferometrically measuring the amplitude and delay of reflected or backscattered light. It stands out because it provides 3D imaging of deep brain tissue at a micron-scale resolution, a reasonable field of view of several millimeters, and feasible temporal resolution, enabling real-time measurements 126. Traditional 3D OCT was invented for ophthalmology with resolution limited to ~10 μm 127. And it has been gradually introduced into cerebrovascular imaging, relying on tracking of blood cells 128. Recent OCT systems can get uniform spatial and axial resolution of 2.48 μm in trophoblast organoids 129. Moreover, OCT endoscopy enables 3D microscopy of the intracranial arterial wall at a resolution approaching 10 µm and a penetration depth of ~ 1 mm 130. With specific microprobe, endoscopic OCT is probably capable of < 3 µm resolution and potentially better imaging contrast 131.

In stroke, the most promising area of OCT is hemodynamics. Optical Doppler tomography (ODT) combined Laser Doppler Flowmeter to OCT, which permits 3D visualization of cerebrovascular networks and quantitative measure for cerebral blood flow (CBF) over a large FOV (e.g., a volume of 2×3 mm^2^ with >1 mm of depth) 132. Pan Y et al. used contrast-enhanced ODT with intralipid to achieve an axial solution of 1.8 μm in visualization of microvascular trees and CBF 133. Optical coherence tomography-based angiography (OCTA) estimates the scattering from moving red blood cells, visualizing functional micro-vessel networks *in vivo* in a rapid, non-invasive fashion 134. Approximately 10 μm resolution can be obtained in visualizing capillaries on the surface of the pial artery 135. It goes beyond Doppler OCT in that spatial and temporal changes in the inner diameter of lumen can be simultaneously monitored by OCTA 135. However, OCTA does not distinguish descending vessels from ascending vessels like ODT. Normalized field autocorrelation function was utilized to resolve it.

### Dynamic light scattering method

#### Laser speckle imaging

Laser speckle imaging (LSI) is a full-field imaging technology for mapping blood flow with high spatio-temporal resolution using simple apparatus, in which areas of higher flow are indicated by lower speckle contrast values 136. LSI can resolve individual blood vessels and provides relative measures of CBF and can be used in cases such as penumbra of cerebral ischemia 137. It has a resolution of 100 μm per se and is rarely used alone for 3D imaging 138. Another limitation is the shallow imaging depth, A normal solution, using microendoscopy, can make a time-lapse blood flow detection in subcortical regions of the brain 139.

It measures changes in blood flow, blood volume, and hemoglobin oxygenation, thus it can be combined with OCT to 3D visualize hemodynamics and oxygenation of vessels at 0.7-0.9mm under the cortical surface at 10μm resolution simultaneously 137. Furthermore, multimodal imaging that combines LSI, OCT, and calcium fluorescence imaging can simultaneously detect cortical hemodynamics, cerebral metabolism, and neuronal activities of the animal brain 140.

#### Diffused optical imaging

Diffuse optical imaging uses NIR to measure physiology millimeters to centimeters deep in the tissue by measuring spatial variations in tissue refractive index and bulk scattering properties. It is used for structural, functional, and metabolic 3D visualization in a low resolution, including neural activity 141, neuronal death, and brain inflammation 142. Diffuse optical tomography (DOT) images changes in cerebral hemoglobin concentration without providing anatomical information. It's powerful in imaging deep, but the resolution is currently stopping at the millimeter level 143, 144. Similarly, diffuse optical correlation tomography (DCT) monitors fluctuation of speckle patterns caused by moving red blood cells to take hemodynamic measurements 145. DCT obtains 3D images of CBF in tissues (~4mm) deep below the cortex in a resolution of ~0.5mm 146. Detection of neuronal cell motion will help DCT achieve sub-second latency 147. DCT combined with LSI allows for probing depths up to ∼10 mm 148.

## 3. Acoustics

### Photoacoustic imaging

Photoacoustic imaging (PAI) combines high contrast based on spectral specificity with the high spatial resolution of ultrasound imaging. PAI can be viewed as ultrasound images whose contrast depends on optical absorption rather than the mechanical properties of tissue 149. In PAI, the most used light is visible light and NIR light with a wavelength between 550 nm and 900 nm. The near-infrared spectrum ranges from 600nm to 900 nm and has a maximum penetration depth of several centimeters 150. Particularly, with the help of contrast agents, the imaging depth can reach 5-6cm 151-153. The theoretical resolution of NIR-PAI is 200 nm, that is, Rayleigh/Abbe diffraction limit 154. However, due to the attenuation effect of photoacoustic, the resolution decreases with the increase of the penetration depth. PAI can be divided into optical-resolution (OR) and acoustic-resolution (AR) PAI. OR-PAI can reach a horizontal resolution of 0.32 mm and a penetration depth of 1mm, while AR-PAI can reach a penetration depth of 3 mm but the resolution is reduced to 45 mm 155. The resolution and penetration depth are like contradictory properties in PAI, while the resolution can be less than 10 nm at depths of several hundred microns, but reduces to submillimeter at depths of centimeters 156. In recent years, the wavelengths of NIR-Ⅱ (wavelength 1000-1700 nm), which allow higher pulse energy, have also been increasingly valued in deep tissue imaging 157. PAI doesn't require cranial windows, and even the use of tiny optical fibers for deep information is minimally invasive 158. Additionally, endogenous tissue chromophores like oxyhemoglobin and melanin provide abundant absorption contrast, for different tissues absorbing light in varying wavelength ranges 159. PAI methods include Photoacoustic tomography (PAT), Photoacoustic microscopy (PAM), Photoacoustic endoscopy, etc., in which PAT is the most common and least restrictive method.

**Figure 5:**
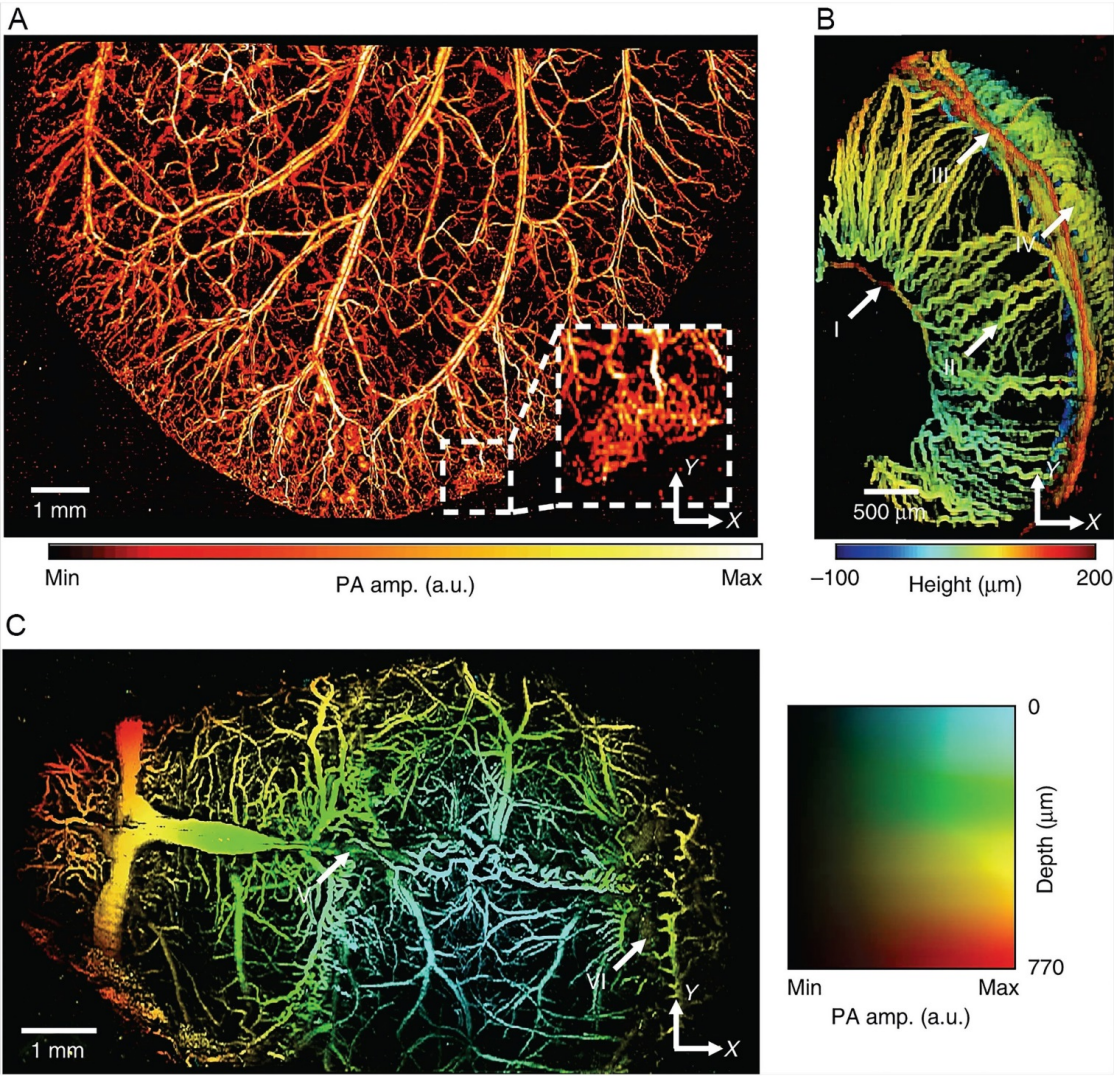
PA images of microvasculatures in small animals in vivo. (A) Wide-FOV PA image of a mouse ear. The region including a capillary bed is outlined by the white dashed box. (B) Depth-encoded PA image of a mouse eye. Circulus arteriosus major (I), iris (II), circulus arteriosus minor (III) and choroid (IV) blood vessels are highlighted by the white arrows. (C) Wide FOV PA MAP image of a mouse brain with color-encoded depths and amplitudes. Superior sagittal sinus (V) and transverse sinus (VI) are highlighted by the white arrows. FOV, field of view; MAP, maximum amplitude projection. Reproduced with permission from Reference 169.

PAI has been adopted to clinical monitoring of stroke 160. In animal models, PAI can be used for structural and functional imaging, but mainly for the latter. Nie L et al. used PAM to observe the microvascular changes in ischemic stroke in the infarcted area at the microscopic level 161. In addition to hemodynamic parameters, oxygen saturation and angiogenesis can be measured by PAI as well 162. The measurement of blood oxygenation is its most important function 163. Song Hu et al. reconstructed the microvascular network in the ears of nude mice with PAM and quantified local oxygen consumption, with an axial resolution of 15 μm 164. Huabei Jiang et al. monitored intracranial hemorrhage in vivo noninvasively by photoacoustic tomography, and the spatial resolution of the system was 180 μm, possibly due to the limitation of attenuation effect 165. The algorithm update can solve this problem. Héctor Estrada et al. corrected the image distortion caused by the PA microscopy due to the skull through the virtual craniotomy deconvolution algorithm and then depicted the 3D skull and vascular anatomical structure 166. The excitation light wavelength was 578 nm, and the resolution could reach 15 μm. The hybrid dual-wavelength PA also improved the axial resolution to 15μm and provided a three-dimensional view of the entire cortical blood vessels of the mice 167. Lihong V Wang's team improved the resolution by using section-assisted photoacoustic microscopy to perform endogenous and natural staining of the mouse brain and cerebellum, showing the three-dimensional distribution of the nucleus with a transverse resolution of 0.91 μm and an axial resolution of about 20 μm 168. But the technology is still in its early stages, and future applications will require it to detect specific endogenous molecules or cells, such as neurons in the brain. Chulhong Kim et al. reported a new positioning PA system with a temporal resolution of 2 ms and a horizontal and vertical resolution of 0.7 and 2.5 μm, respectively **(Figure 5)** 169. The new PAI technology can already display multiple hemodynamic photoacoustic data noninvasively in real time, but the spatial resolution is only 200μm, which misses microvascular system 170. Nevertheless, it shows great potential in the field of stroke, especially neuroimaging 171. Neuroimaging of PA can also be based on hemoglobin gradient and oxygen saturation changes, but the resolution is limited to several hundred microns 172. GCaMP is primarily used, while it cannot be used to image neurons deep in the brains of living mammals 173, 174.

## 4. Magnetic resonance imaging

Magnetic resonance imaging (MRI) is best known for its extensive use in clinical practice. MRI is an indispensable imaging method for the evaluation of cerebral small-vessel diseases, such as microstructural changes in white matter and gray matter, microhemorrhage near small arteries, and subcortical infarction of the cerebellum 175. MRI is mostly done by measuring the radio frequency signals emitted by hydrogen atoms after applying particular radio frequency (RF) waves from RF coils, and positioning the signals using spatially varying magnetic gradients for noninvasive imaging, while also avoiding radiation damage by providing 3D information of soft tissues from water content. The contrast of each voxel depends on the proton density in the voxel and the properties of the local tissue microenvironment 176. Conventional MRI is primarily used for anatomical and functional imaging of cm-scale samples with a spatial resolution of about 1 mm because its resolution is constrained by the inherent low signal-to-noise ratio (SNR) of MRI 177. Higher magnetic field intensity leads to higher spatial resolution, but the related increase in spectral line width is a necessary factor to be considered 178. Therefore, the high SNR and strong gradient required for accurate MRI datasets determine that it is more suitable for ultra-high-field imaging, which avoids deviation from near field compared with low field 179. Professor Budinger is advocating raising the magnetic field from 14 T to 20 T, highlighting the benefits of the technique for studying the human brain 180. The high and ultra-high fields do bring high resolution, with an amazing 100 nm resolution of 3D imaging on a large sample such as the whole brain 181. However, it cannot cater to the smaller micron scale structure. In fact, micron-scale 3D MRI images have been available for more than ten years. L.Ciobanu used a spatial resolution of 3.7×3.3×3.3 μm^3^ to image a single cell and observed the helical arrangement of chloroplast 182. Several methods have emerged in recent years to improve the resolution of MRI—a previously considered "macro-imaging" technique- for studies of micron neurovascular structures in brain in stroke.

MRI contrast agent is feasible to improve the resolution. The contrast agent can be distributed to extracellular fluid, blood vessels, and even specific organs. By shortening T1 or T2 relaxation time, the signal intensity on T1-weighted images increases or the signal intensity on the T2-weighted image decreases 183. T1-weighted contrast agents for brighter signals are more suitable for higher resolution Paramagnetic contrast agents except dysprosium compounds are positive contrast agents; and the superparamagnetic or ferromagnetic materials that form negative contrast agents can also change particle size or coating to become positive contrast agents 184, 185. Gadobutrol, a kind of contrast agent, was used to enhance the resolution of MRI in observing post-stroke brain edema and achieved an isotropic spatial resolution of 100 μm 186. The application of the contrast agent Magnevist (a kind of gadolinium chelate) can obtain 3D imaging of structures such as hippocampus at 40μm resolution 187. Manganese enhanced magnetic resonance imaging (MEMRI) can be used for both functional and structural imaging. Paramagnetic manganese ions mimic Ca^2+^ and accumulate in neurons through Ca^2+^ channels and are therefore used to study neuronal pathways or pathophysiological processes in stroke 188. Chika Sato et al. improved MEMRI technology in vitro and obtained isotropic 3D neuroimaging with 25 μm resolution using low temperature RF coils 189.

Low temperature RF technology is equally vital in improving resolution. At low temperatures of tens of degrees Kelvin, the strength of the nuclear magnetic resonance signal can be enhanced by a large spin polarization at thermal equilibrium, thus reducing thermal noise. Meanwhile, dynamic nuclear polarization (DNP) can transfer polarization from electron spin to nuclear spin, and finally, obtain a resolution better than 1 μm 190, 191. The whole brain MRI scan was reduced to 11 minutes, showing both nerve and vascular lesions at the same time 191. Robert Tycko et al. obtained the ^1^H MRI image with an isotropic resolution of 2.8 µm in glycerin/water at 28 K 192. The low temperature also fulfilled high quality ^13^C and ^39^K MRI images for metabolic studies 193, 194.

In addition to the relaxation of T1 and T2, MRI can be based on another contrast mechanism—Brownian motion of water molecules, including diffusion weighted imaging (DWI) and more advanced diffusion tensor imaging (DTI), and their grayscale pixel values depend on the basic diffusivity of these voxels 195. DWI and DTI have been widely used in the clinical diagnosis and research of brain diseases, including cerebral small vascular disease 196. DWI can detect the differential distribution of apparent dispersion coefficient (ADC) 197. The higher the ADC values, the stronger the dispersibility of water molecules is. Choong H. Lee et al. achieved an isotropic spatial resolution of 10μm of drosophila brain using DWI with RF microsurface coils 198. The research conducted by Chen et al. utilized DWI in human and porcine adjacent α-neurons also obtained 3D reconstructed images with an isotropic resolution of 6.25 μm 199. The DTI estimates the degree of diffusion anisotropy along the direction of the main fibers, producing features such as mean diffusivity and fractional anisotropy, thus can characterize the neural structures of gray and white matter, but with relatively low resolution 200, 201. DWI is very sensitive to motion, and it is inevitable to prolong scanning time while avoiding ghosting, but for DTI, the large amount of diffusion directions required often means to sacrifice spatial resolution 195.

## 5. Conclusions and perspectives

The physiological process of stroke is based on the tight and orchestrated relationship between nerves and vasculature. Nerves are closely related to the surrounding blood vessels. The concept “neurovascular unit” (NVU) focused on the coupling between neural activity and blood flow to explain how neuronal signals modulate nearby microvessels to support cerebral function 202. Meanwhile, the molecular pathways shared by the neurons and vessels play a role in vascular influence on the homeostasis of the nervous system 203. Therefore, neurovascular structural and functional imaging is important to explore the physiological or pathological processes of stroke. Over decades of years, many 3D imaging technologies have been developed or improved in resolution. But the need for further research still raises questions about these techniques.

In order to conform to the physiological state, it is necessary to study the nerve and blood activity in the waking state. Fluorescence-labeled TPM and LSM require preparation of cranial windows and are therefore invasive. PAI and MRI are noninvasive. Updates to traditional methods, such as μCT with blood-pool liposomal-iodinated contrast agent 204, fluorescence imaging through a near-infrared window 205, and ultrasound microscopy with special contrast agents 206, also yielded noninvasive, high-resolution three-dimensional images, but mainly for cerebral blood vessels. Observation of living bodies is always limited to shallow structures, while deeper structures or observations of large animals such as humans require deeper penetration. Cumulative scattering limits the penetration depth of optical microscopy. The depth of TPM is stronger than that of LSM, which can reach 1mm. Through the near-infrared window, many optical microscopy techniques have improved the penetration depth and the maximum penetration depth is about 3mm 205. However, it can still only observe the superficial nerves and vessels. PAI can penetrate several centimeters deep, but it severely sacrifices resolution. MRI and CT that can obtain the structure of the whole brain are difficult to obtain micron resolution. Optical fiber is a viable attempt to achieve multiple penetration depths in both TPM and LSM. Further, we are pursuing the imaging of free-moving animals. The wearable PAT can only achieve a spatial resolution of 200µm, while high-resolution hemodynamic observations lack 3D imaging results 207, 208. At a micron-resolution scale, tiny motions can also greatly affect image quality. TPM is a more feasible approach that can simultaneously image multiple functional areas of the brain, but it tends to study signals in neural circuits 209-211. Thus, it is necessary to make a comparison of these imaging techniques **(Table 1)**.

**Figure 6:**
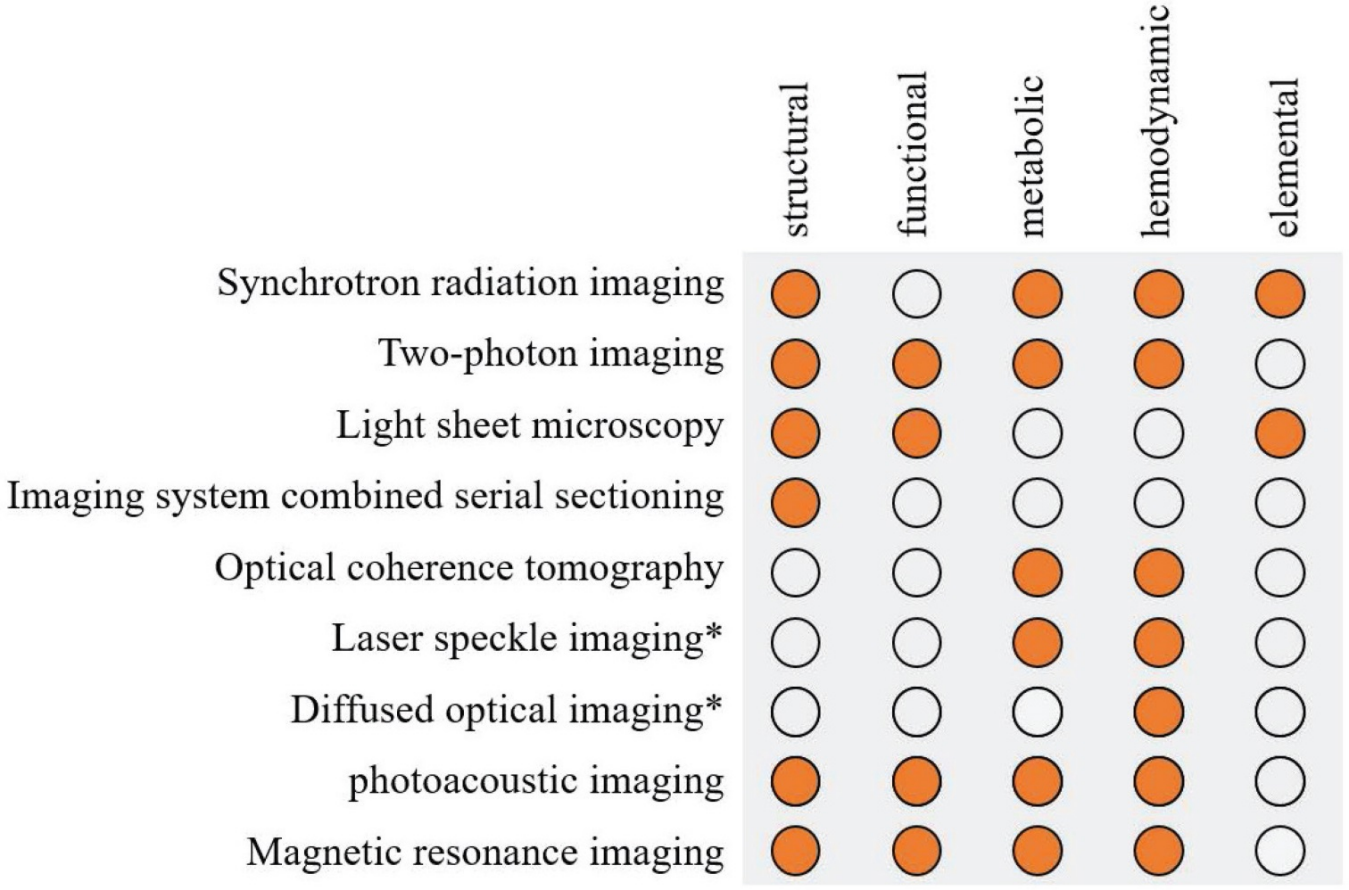
Summary of the application of high resolution imaging method in stroke. Solid orange circles indicate the use of this technology. *Laser speckle imaging and diffused optical imaging are low-resolution imaging methods but can be combined with other structural imaging to obtain high resolution.

With the diversification of detection indexes and the refinement of detection, multi-mode imaging technology combining complementary microscopy techniques has been developed. PAI, which stands for multimodal, is a combination of photoexcitation and acoustic detection, maintaining high resolution at deeper imaging depths. Combining with CT is a common multi-mode method to improve resolution, such as SR-CT and photoacoustic computed tomography (PACT) 212. Multi-modality also provides richer detection results, for example, combining the functional information of DOT with a high resolution structural imaging will achieve a high resolution functional imaging 213. LSM combined with nonlinear two-photon excitation can improve the imaging depth and speed 85. Fluorescence imaging has been applied in a great assortment of modalities (e.g., PAT and MRI) to study molecular events, thus makes a difference in structural, functional or molecular imaging 214. Figure 6 summaries the application of high resolution imaging method.

It is worth noting that there are currently several techniques that play an important role in functional, hemodynamic, and metabolic imaging, but fail to achieve micron resolution, such as functional near-infrared spectroscopy (fNIRS) 215, intrinsic signal optical imaging (ISOI) 216, and single-photon emission computed tomography (SPECT) 217. However, diffused optical imaging is also a low resolution technique, but it achieves high resolution through multimodality.

Optical microscopy is obviously the most important of the above techniques based on different principles, but its resolution is always limited to the scattering limit. In recent years, the development of ultramicroscopy with super-resolution facilitates the resolution beyond the scattering limit and can reveal the landscape of the cellular organelle interactome or observe small intact animals 218, 219. It has provided innovation from the micron level to the nanoscale level, which is introduced briefly in the part of LSM. Of course, it is used in PAI, TPM, and other technologies as well 220, 221. It can focus our attention on more subtle structures, but as is discussed earlier, we have both nanoscale and macroscopic means of observation, while ignoring spatial relationships on the micron level; we have a wealth of techniques for monitoring nerves and vessels separately, but we still cannot observe and interpret neurovascular coupling well. At the same time, we are increasingly aware of the close relationship between nerves and vasculature in stroke, thus high-resolution 3D imaging may inevitably become the key to further research.

## Figures and Tables

**Table 1:** Advanced 3D imaging technology and characterizations in imaging of small mammals.

Advanced 3D imaging technology		Focus point			
		Whole brain scale	Imaging depth	Living animals	Behaving animals
Light	Synchrotron radiation imaging	√		Absorption imaging	
	Two-photon imaging		up to mm-level	√	√
	Light sheet microscopy		up to mm-level	some methods	some methods like SCAPE
	Imaging system combined serial sectioning	√			
	Optical coherence tomography		up to mm-level	√	√
	Dynamic light scattering method		up to mm-level	√	√
Acoustics	Photoacoustic imaging		up to cm-level	√	√
Magnetics	MRI	√		√	√

Multimodal imaging: combined with the different techniques in the table above, or super-resolution microscopy, etc. On the right side of the table are some separate focus points for imaging the brains of animal models, particularly rodents, including whether they are used for whole-brain imaging, the depth of imaging, and whether they are used in living or behaving animals. SCAPE: swept, confocally-aligned planar excitation. μCT: microcomputed tomography.

## Abbreviations

3D: three-dimensional
MRI: magnetic resonance imaging
SR: synchrotron radiation
APS: American Light Source
ESRF: European Synchrotron Radiation Facility
SLS: Swiss Light Source
SSRF: Shanghai Synchrotron Radiation Facility (SSRF)
KESA: K‐edge subtraction angiography
SETS: single‐energy temporal subtraction
PCI: Phase contrast imaging
ILPCI: inline X-ray phase contrast imaging
GI: grating interferometry
CI: crystal interferometry
ACI: absorption contrast imaging
MCAO: middle cerebral artery occlusion
µCT: microcomputed tomography
FOV: fieldof view
SR-IR: SR-infrared spectrumor
SR-μXRF: SR micro-X-ray fluorescence
FTIR-μCT: fourier transform infrared spectro-microtomography
TPM: two-photon microscopy
NIR: near-infrared
MPM: multi-photon microscopy
ChroMS: chromatic multiphoton serial
GRIN: gradient-index
AOS: adaptiveoptics systems
LSM: light-sheet microscopy
SPIM: Selective Plane Illumination Microscope
LLSM: lattice light-sheet microscope
SCAPE: swept confocally-aligned planar excitation
FITC: Fluorescein isothiocyanate
Mars-SPIM: multiangle-resolved subvoxel selective plane illumination microscope
anti-αSMA: anti-αsmooth muscle actin
MOST: micro-optical sectioning tomography
fMOST: fluorescent micro-optical sectioning tomography
STP: serial two-photon tomography
FAST: block-face serial microscopy
GFP: green fluorescent protein
OCT: optical coherence tomograph
ODT: optical Doppler tomography
CBF: cerebral blood flow
OCTA: optical coherence tomography-based angiography
LSI: laser speckle imaging
DOT: diffuse optical tomography
DCT: diffuse optical correlation tomography
PAI: photoacoustic imaging
OR: optical-resolution
AR: acoustic-resolution
PAT: photoacoustic tomography
PAM: photoacoustic microscopy
MAP: maximum amplitude projection
RF: radio frequency
SNR: signal-to-noise ratio
MEMRI: Manganese enhanced magnetic resonance imaging
DNP: dynamic nuclear polarization
DWI: diffusion weighted imaging
DTI: diffusion tensor imaging
ADC: apparent dispersion coefficient
NVU: neurovascular unit
PACT: photoacoustic computed tomography
fNIRS: functional near-infrared spectroscopy
ISOI: intrinsic signal optical imaging
SPECT: single-photon emissioncomputed tomography
